# Optimizing Excipient Properties to Prevent Aggregation in Biopharmaceutical Formulations

**DOI:** 10.1021/acs.jcim.3c01898

**Published:** 2023-12-19

**Authors:** Toby E. King, James R. Humphrey, Charles A. Laughton, Neil R. Thomas, Jonathan D. Hirst

**Affiliations:** † Biodiscovery Institute, School of Pharmacy, University Park, Nottingham NG7 2RD, U.K.; ‡ Croda Europe Ltd, Cowick Hall, Snaith DN14 9AA, U.K.; § Biodiscovery Institute, School of Chemistry, University Park, Nottingham NG7 2RD, U.K.; ∥ School of Chemistry, University Park, Nottingham NG7 2RD, U.K.

## Abstract

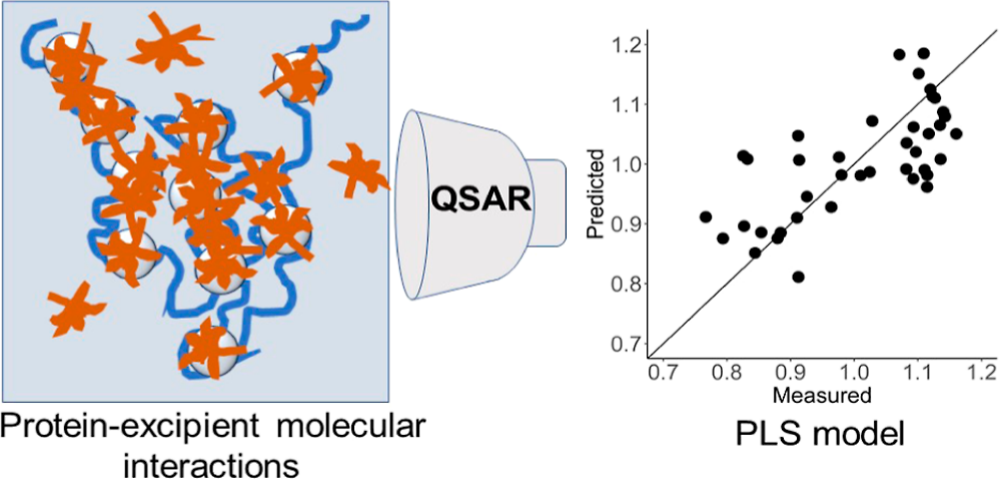

Excipients are included within protein biotherapeutic solution formulations to improve colloidal and conformational stability but are generally not designed for the specific purpose of preventing aggregation and improving cryoprotection in solution. In this work, we have explored the relationship between the structure and antiaggregation activity of excipients by utilizing coarse-grained molecular dynamics modeling of protein–excipient interaction. We have studied human serum albumin as a model protein, and we report the interaction of 41 excipients (polysorbates, fatty alcohol ethoxylates, fatty acid ethoxylates, phospholipids, glucosides, amino acids, and others) in terms of the reduction of solvent accessible surface area of aggregation-prone regions, proposed as a mechanism of aggregation prevention. Polyoxyethylene sorbitan had the greatest degree of interaction with aggregation-prone regions, decreasing the solvent accessible surface area of APRs by 20.7 nm^2^ (40.1%). Physicochemical descriptors generated by Mordred are employed to probe the structure–property relationship using partial least-squares regression. A leave-one-out cross-validated model had a root-mean-square error of prediction of 4.1 nm^2^ and a mean relative error of prediction of 0.077. Generally, longer molecules with a large number of alcohol-terminated PEG units tended to interact more, with qualitatively different protein interactions, wrapping around the protein. Shorter or less ethoxylated compounds tend to form hemimicellar clusters at the protein surface. We propose that an improved design would feature many short chains of 5 to 10 PEG units in many distinct branches and at least some hydrophobic content in the form of medium-length or greater aliphatic chains (i.e., six or more carbon atoms). The combination of molecular dynamics simulation and quantitative modeling is an important first step in an all-purpose protein-independent model for the computer-aided design of stabilizing excipients.

## Introduction

Protein-based biotherapeutics are a growing market, with significantly more treatment options based on biologics under development and a multibillion dollar industry revolving around their research and manufacture; in 2021, 28% of all FDA-approved drugs were biologics.^1^ The majority of biotherapeutics include hormones,^2^ plasma proteins,^3^ enzymes,^4^ coagulation factors,^5^ vaccines,^6^ and monoclonal antibodies (mAb) and their fragments.^7^ mAbs are the largest fraction^8^ and are used primarily as immunotherapeutics, for targeted delivery,^9^ and cancer vaccines.^10^ Generally, therapeutic proteins are produced in bioreactors using recombinant cell lines^11^ and are often lyophilized or frozen for storage. One of the key challenges facing protein biotherapeutics is their conformational and colloidal stability as formulation and storage conditions can induce aggregation and agglomeration^12^ during both freezing and rethawing or resuspension.^1^ These aggregates have reduced function^13^ and an increased specific immune response when administered;^14^ indeed, the association constant of human serum albumin (HSA) to ketuprofen decreased by 42% after the formation of fibrillar aggregates by HSA.^15^

**Figure 1:**
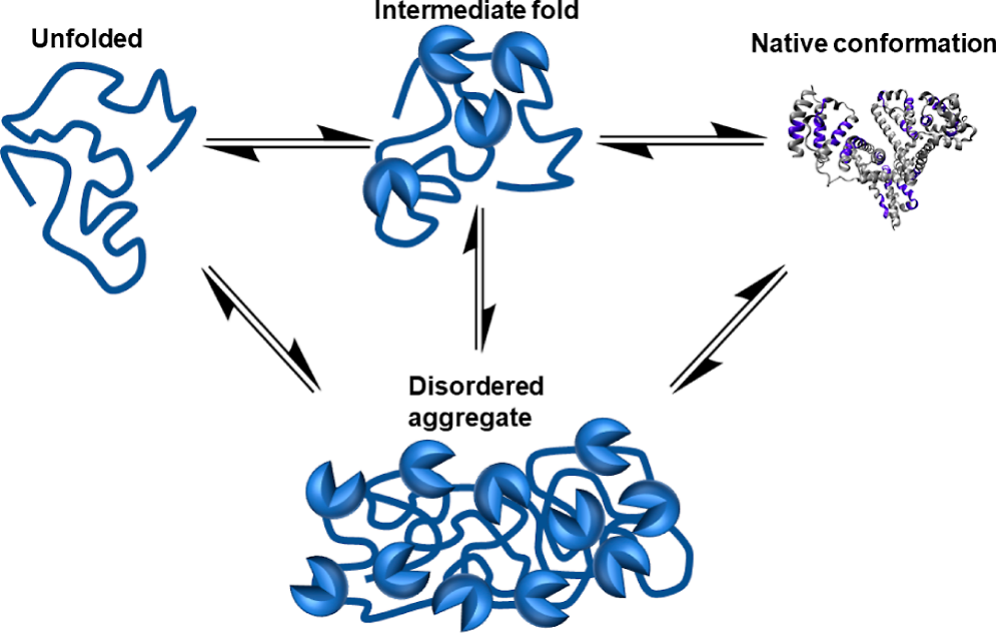
Folding and misfolding pathways of a protein. An unfolded protein assumes more stable intermediate folding conformations until arriving at the native configuration. If subjected to non-native conditions, the equilibrium position can change to favor the formation of a stable, disordered conformation, which can form an aggregation nucleus while residing within a thermodynamic energy minimum.

As folding occurs, the tertiary structure of a protein changes as hydrophobic residues are buried within the 3D structure. The folding protein assumes transient intermediate structures of increasing stability and reaches a thermodynamic global minimum at the native conformation, sometimes guided by molecular chaperone proteins.^4,13,16^ During manufacture and storage, proteins are exposed to non-native conditions, such as nonphysiological pH, ionic strength, extremes of temperature, interactions with impurities, and hydrophobic interactions at interfaces with synthetic surfaces or air, which may induce partial unfolding or misfolding and can lead to noncovalent aggregation (Figure 1). The change in the structure may expose hydrophobic residues, which form patches on the surface of the protein.^17^ The energy landscape changes; it becomes more favorable to bury the hydrophobic patches by interaction with hydrophobic surfaces, such as similar patches on other protein molecules. This process is driven primarily by hydrophobic interaction, but electrostatics and hydrogen bonding also contribute.^13^ Solvent is preferentially excluded from the protein surface as the protein molecules interact with one another, and more molecules are recruited into the aggregation nucleus in an irreversible process.^18^

The tendency of protein biotherapeutics to aggregate can be mitigated by the modification of conditions, such as pH^19^ and ionic strength,^20^ as well as the inclusion of excipients into biotherapeutic formulation.^12,21^ Excipients used to improve biotherapeutic stability include histidine,^22^ arginine,^23^ sugars,^24^ fatty alcohol ethoxylates,^25^ alkylsaccharides,^26^ poloxamers,^27^ and polysorbates.^28^ The mechanism by which aggregation is prevented is not fully understood. One proposal is the formation of protein–excipient complexes, which could shield aggregation-prone regions (APRs) of the protein from solvent or other hydrophobic surfaces.^29,30^ Competitive adsorption at surface interfaces, particularly by surfactants, may prevent aggregation by reducing the exposure of the protein to another hydrophobic surface, thereby reducing partial unfolding and aggregation nucleation.^31,32^ Excipients also modify the energetics of native intermediates and increase stability, by making disordered intermediates less favorable and acting as a chaperone to facilitate native folding.^33^

Differences in the protein structure complicate the understanding of aggregation prevention; as proteins’ structures differ, so too will their aggregation propensity, as well as their interaction with antiaggregation agents. Hydrophobic patches of proteins are exposed to solvent to different extents, and APRs will not have the same topology and charge distribution across different proteins.^17^ There are multiple approaches to predicting APRs using differing levels of the protein structure. Some, such as Aggrescan,^17^ work solely from the primary structure and determine APRs by comparing the amino acid sequence against an experimentally determined aggregation propensity. Others account for the 3D structure and, thus, the solvent-accessible surface area (SASA). Examples of this approach include SAP^17^ and Aggrescan-3D.^34^ Generally, excipients are chosen not in light of efficacy as an antiaggregation agent, but due to their well-established safety profiles from other uses;^35^ for example, polysorbates are popular emulsifiers, particularly in cosmetics^36^ and in the food industry.^37^ Therefore, there is chemical space to explore to optimize antiaggregation excipients.

Computational techniques can provide mechanistic insights into length and time scales that are inaccessible to conventional wet lab methods.^38^ Molecular dynamics (MD) simulations have been applied in the study of surfactant behavior in solution;^39,40^ protein–surfactant interaction,^29^ including stability modulation^41^ and binding;^42^ protein aggregation^43^ and folding;^44^ and the modulation of protein stability by excipients such as histidine.^45^ Atomistic or pseudoatomistic MD models often have a prohibitively high computational expense to be applied in large numbers of simulations that examine microsecond-time scale events, such as many aspects of protein dynamics.^46^

There are few investigations of the nonspecific interaction between excipients and APRs as a mechanism of aggregation prevention that considers all areas of the protein. No quantitative structure–property relationship model has been derived that probes the relationship between the excipient structure and antiaggregation activity. In this work, we present an MD model that investigates APR–excipient interaction to determine the stabilizing effect on protein biotherapeutics, coupled with a quantitative model which uses physicochemical descriptors in statistical analysis to reveal the impact of the key features on antiaggregation activity. In doing so, we investigate the model of the shielding of APRs from solvent as a mechanism of aggregation prevention, hypothesizing that a smaller SASA of APRs leads to greater stability. To produce sufficient data for a quantitative model, a coarse-grained (CG) force field was selected, as they allow access to microsecond simulation time scales at reasonable computational expense and without the need for enhanced sampling methods. CG force fields decrease the computational cost at the expense of resolution by representing multiple atoms as a single interaction site; doing so can facilitate the large-scale simulation at microsecond time scales, as there are fewer degrees of freedom to consider.

MARTINI^47,48^ is a prominent CG force field which maps atoms to beads at an approximately 4:1 ratio in a building-block approach. It has been applied to many different biomolecular systems, such as membrane studies, protein–ligand binding, phase behavior, carbohydrates, and nucleic acids. MARTINI has also been applied specifically in the context of improving protein stability by including excipients that reduce antibody self-association; Lui et al. utilized a docking approach to screen excipients by binding with the most significant APR. The Docking Assay For Transmembrane components (DAFT) method for the high-throughput study of dimer/trimer association^49^ was applied in order to sample sufficient initial relative poses of antibody fragments, resulting in a CG-MD model of antibody self-association and the effect of excipients on aggregation kinetics.^50^ Similarly, insulin self-association and its non-Arrhenius behavior were investigated in a study of aggregation nucleation kinetics in MARTINI,^51^ finding that the insulin unfolding equilibration constant is the single most important kinetic parameter in nucleation time.

Excipients were selected based on their prevalence in the industry as solution state stability enhancers, their prior parametrization by the MARTINI development team, or their utility to a quantitative model. PEG alkyl amides (PAAs) consist of a PEG chain, amide linker, and alkyl chain. Fatty acid ethoxylates (FAEs) and fatty alcohol ethoxylates compounds are similar but have an ester bond or an ether bond in place of the amide linker, respectively. Polysorbates are fatty acid esters of polyoxyethylene sorbitan (PSBN). Spans are similar to polysorbates but are not ethoxylated.^52^ Other compounds include cholesteryl glucopyranoside, a range of phospholipids, fatty acids, arginine, and *n*-octyl glucoside. This range of chemically diverse compounds facilitates the extraction of useful information for quantitative modeling and allows data-driven decisions to be made in the design of antiaggregation excipients. The application of these data could improve biotherapeutic formulation design by lowering costs, improving therapeutic outcomes, and elucidating structure–property relationships.

HSA was chosen as a model protein due to its use in biotherapeutic formulations, both as an active pharmaceutical ingredient^53^ and an excipient,^54^ its loss of function after aggregation,^15^ and its manageable size of 585 residues. Some evidence indicates that the binding between HSA and excipients (specifically polysorbates) occurs within endogenous binding sites^55^ and thus could pose difficulties in extrapolating the model to other therapeutically relevant proteins, particularly as the same study indicated negligible interaction between polysorbates and IgG. However, there is also evidence of polysorbates interacting with pharmaceutically relevant proteins, including human growth hormone,^56^ an IgG mAb,^57^ filgrastim,^58,59^ lysozyme, RN295, and recombinant factor VIII,^60^ imparting improvements to their physical stability, in conjunction with surfactant and interfacial stabilizing interactions. Thus, the interaction between HSA and the excipients selected for this study could feasibly be applied to different proteins to elucidate excipient and protein interactions and their potential roles in preventing aggregation.

## Materials and Methods

The initial structure of HSA was obtained from the RCSB Protein Data Bank (code 4L8U)^61^ and processed into the MARTINI force field via the martinize2 script, from the vermouth package.^62^ Its APRs were highlighted using the Aggrescan web server^17^ and its FASTA sequence; the APRs consisted of 25.4% of the sequence in 18 different patches.

### Parameterization

**Figure 2:**
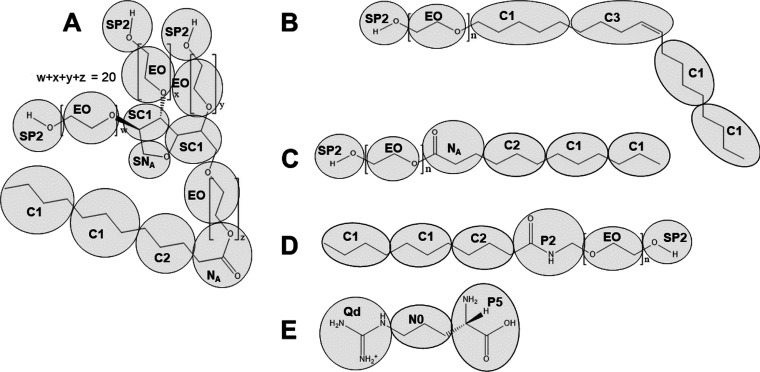
Chemical structures of studied excipients with their MARTINI mapping superimposed. (A) Polysorbate 20, (B) fatty alcohol ethoxylate (Brij) O*n*, (C) FAE L*n*, (D) PEG alkyl amide L*n*, and (E) l-arginine

To parametrize excipients that are not available from MARTINI, initial united-atom coordinates and topologies were generated using the Automated Topology Builder^63^ in the GROMOS 54a7 force field^64^ and converted into a MARTINI model. The MARTINI mapping was based on existing MARTINI beads and their use in the literature, as well as the preservation and representation of functional groups (Figure 2). Molecule parameters reported in the previous work by the MARTINI group and used here include phospholipids, ceramides, and glycerols,^65^ as well as sugars,^66^ fatty acids,^67^ and sterol groups.^68^

The initial united-atom structure is simulated for 10 ns in water at pH 7.0 and indexed so that each index group of atoms corresponds to a MARTINI bead. The angles and distances between these beads are measured and used as the bonded parameters in the MARTINI topology, a frame is extracted and used as the initial structure for a MARTINI simulation, and the bond lengths and angles are measured. These values and their force constants are modified in an iterative process until their distributions throughout both the MARTINI and indexed simulations are approximately matched. Polyply^69^ was also used to generate initial MARTINI topologies for some compounds.

### Molecular Dynamics Simulation

All MD simulations were carried out using GROMACS 2019 and 2021.2 in the Martini 2.3P force field, and five independent simulations were performed for each system. A truncated octahedral box was built around a single molecule of HSA, with the distance between opposing hexagonal sides equaling 34.24 nm, leading in practice to a volume of 30841.5 nm^3^. Each simulation contained a single molecule of HSA and approximately 233,000 MARTINI water molecules, for a protein concentration of 0.0538 mM or 3.61 mg/mL; therapeutic HSA formulations are typically between 5 and 25% w/v.^70^ This size was a compromise between having a sufficiently large system to model a comparatively low excipient concentration, with enough excipient molecules for the system to be thermodynamically realistic, and the prohibitive computational expense that would result from larger systems to model concentrations approaching those found in HSA therapeutic formulations. Sufficient excipient molecules were added to bring their concentration to 0.1% w/w, an industrially relevant concentration^71,72^ via gmx insert-molecules inserting into vacuum. In practice, this leads to a variable molar concentration, proportional to the molecular weight of the excipient. This is not an issue as it maintains the quantity of Martini “beads” across all simulations and makes comparisons between them more straightforward. The vacuum system was minimized for 1000 steps using the steepest descent algorithm and solvated using the MARTINI polarizable water model.^73^ Sodium ions were added to neutralize the system by replacing water molecules at random, and the system was minimized again for 1000 steps. The system is relaxed in the *NPT* ensemble, with a 5 fs time step, V-scale thermostat at 300 K, and isotropic pressure coupling at 1.0 bar using the Berendsen barostat.^74^ This relaxation phase consisted of 100 ps. Production MD was performed in the same ensemble, with the same thermo- and barostats, a time step of 20 fs, and a total time of 1 μs. For some compounds, particularly those with ring structures, a time step of 10 fs was necessary to run stable MD; the overall time remained 1 μs. Coulombic and Lennard-Jones cutoffs were 1.1 nm and used the reaction field and potential shift Verlet modifiers, respectively, in the Verlet cutoff scheme. All trajectories were found to be equilibrated and converged, which in detail can be found in the Supporting Information. Full parameter files can be found in the Github repository (see Supporting Information).

The SASA of the APRs was calculated using gmx sasa within Gromacs, indexed to calculate the SASA of APRs alone, using lone HSA as a control. Bartlett’s test^75^ was utilized to indicate homoscedasticity between distributions for each excipient–protein simulation, and the results directed whether the Kruskal–Wallis^76^ (homoscedastic) or Welch’s^77^ (heteroscedastic) analyses of variance were employed to determine statistical significance. All analysis scripts can be found in the GitHub repository (Supporting Information).

### Structure–Property Relationship

To probe the structure–property relationship of antiaggregation activity, partial least-squares (PLS)^78^ regression was performed, using a set of physicochemical descriptors as input. Molecular descriptors were generated using the Mordred package^79^ in Python and filtered based on their utility in the context of chemical intuition, leaving a total of 106 descriptors. PLS regression was performed on the entire data set, employing leave-one-out cross validation^80^ (LOO-CV) to find the optimal number of components to include in the model. This is achieved by using a number of components that cause the root mean squared error of prediction to be at a minimum, while also taking into account the principle of parsimony and avoiding overfitting. Four components were used in the final PLS model. To measure the robustness and efficacy of the model in predicting data, the data set was split into a partition of 0.8/0.2 training data/test data. LOO-CV was performed on the training data set, the model was applied to predict the test data set, and the *Q*^2^ was recorded as a measure of predictive accuracy. This was repeated 1000 times; the *Q*^2^ reported hereafter is the median of these repetitions.

## Results

### Protein–Excipient Interaction

**Figure 3:**
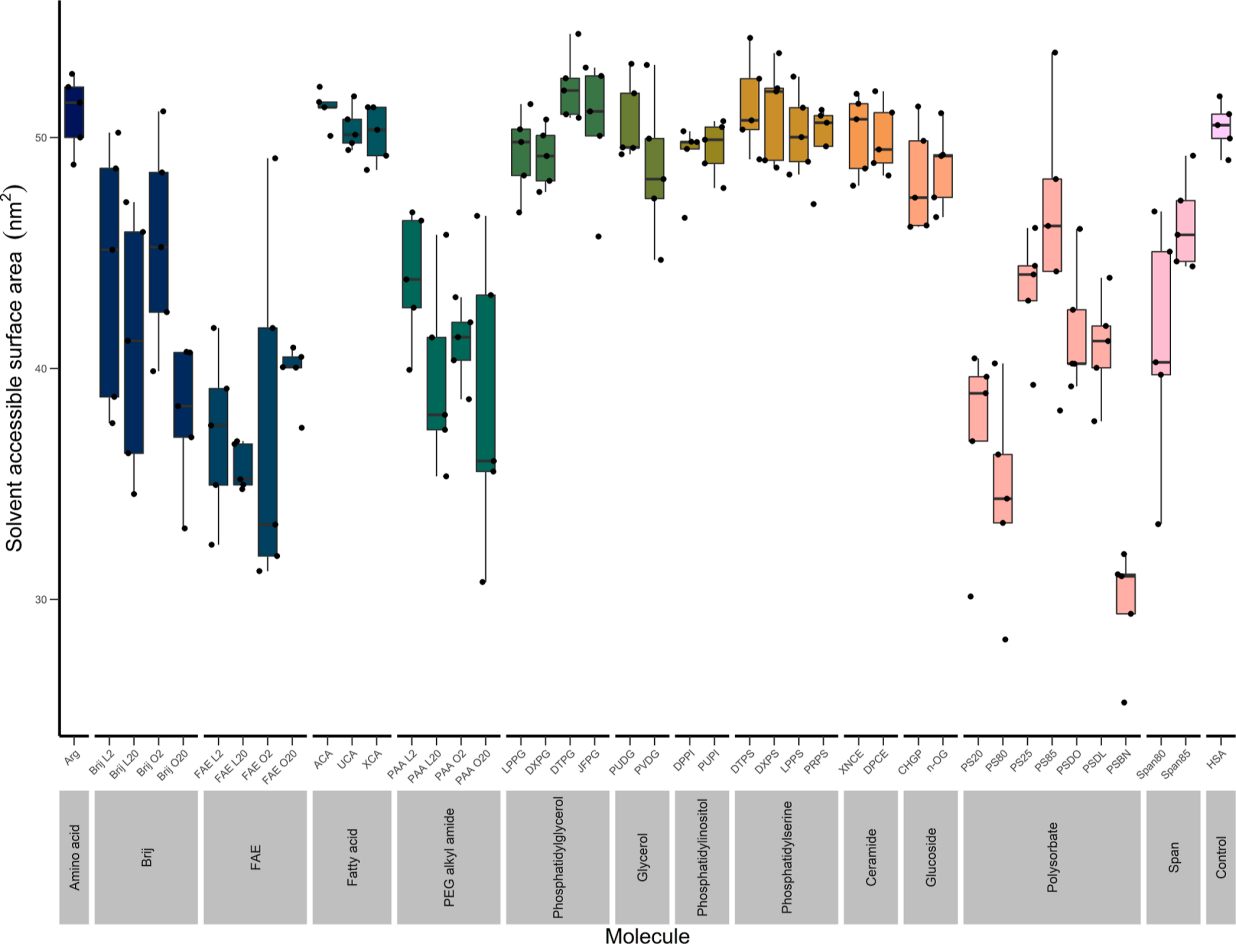
Average SASA of APRs, averaged for each trajectory. Polysorbates have the greatest effect on the SASA of APRs. Of the linear, ethoxylated surfactants, there is little significant difference between them, across all classes, but they are all significantly different from the control, with the exceptions of Brij O2 and L2. Arginine, phospholipids, fatty acids, and glucosides had an insignificant effect on the SASA of the APRs.

The shielding of APRs from solvent by excipient molecules is a key mechanism in the prevention of aggregation and increase in stability of biotherapeutic protein formulations; this can be quantified in an MD model by the extent to which the SASA of APRs reduces. HSA without any excipients was found to have a SASA of 271.7 nm^2^; within that, its APRs have an average SASA of 50.5 nm^2^ polysorbate compounds have the greatest impact on the SASA of APRs (Figure 3) and are all statistically significant from the HSA-only control, according to Kruskal–Wallis^76^ and Dunn tests. PSBN, the strongest performer, is significantly different from Brij L2 (*p* < 0.05), Brij O2 (*p* < 0.05), and PS85 (*p* < 0.01). PS80 is significantly different from Brij O2 (*p* < 0.05), which is somewhat surprising, given that they contain the same aliphatic chain content (a single oleate). Linear ethoxylated compounds were not significantly different from one another, with the exception of Brij O2, which was different from every other linear ethoxylated compound. (*p* < 0.05). The only ethoxylated compounds to not be significantly different from the control were Brij O2, Brij L2, and Span 85. Span 80 was significantly different from the control, but no difference was found between it and any polysorbate compound. None of the other compounds under study were found to have an impact on the SASA of the APRs of HSA that was significantly different from the control.

There is a significant degree of heterogeneity in performance, within both a single class and repetitions of the same excipient. This could be indicative of the nonspecific nature of binding; the interaction within each individual repetition and each individual molecule could be between many different residues in a heterogeneous manner, and a weak interaction might not guarantee the formation of an HSA-excipient complex within the simulation time. In each trajectory, protein–excipient contacts remained dynamic to some degree, fluctuating above and below the average. Each trajectory appeared to be at equilibrium in this way. This is indicative of the interaction being somewhat reversible, although the deviation from the average throughout a given trajectory is not large.

The significant α-helical content of HSA will have an effect, as the configuration in space will affect both the accessibility of specific residues and the local environment in which they reside. This is represented in MARTINI as a change in the polarity of the backbone bead of all residues present in a helix as well as the side chains of glycine, alanine, and protein, all represented as significantly less polar beads.^81^ Therefore, an alanine residue within an α-helix will have significantly less hydrophobic character than an alanine residue outside a helix. As 87.5% of the APRs are found within α-helices and 39.1% of the residues within the helices are APRs, it is likely that the interaction between helices and excipient or between helices and solvent is significant in aggregation prevention. Indeed, α-helices have been shown to induce the formation of protein aggregates.^82,83^

**Figure 4:**
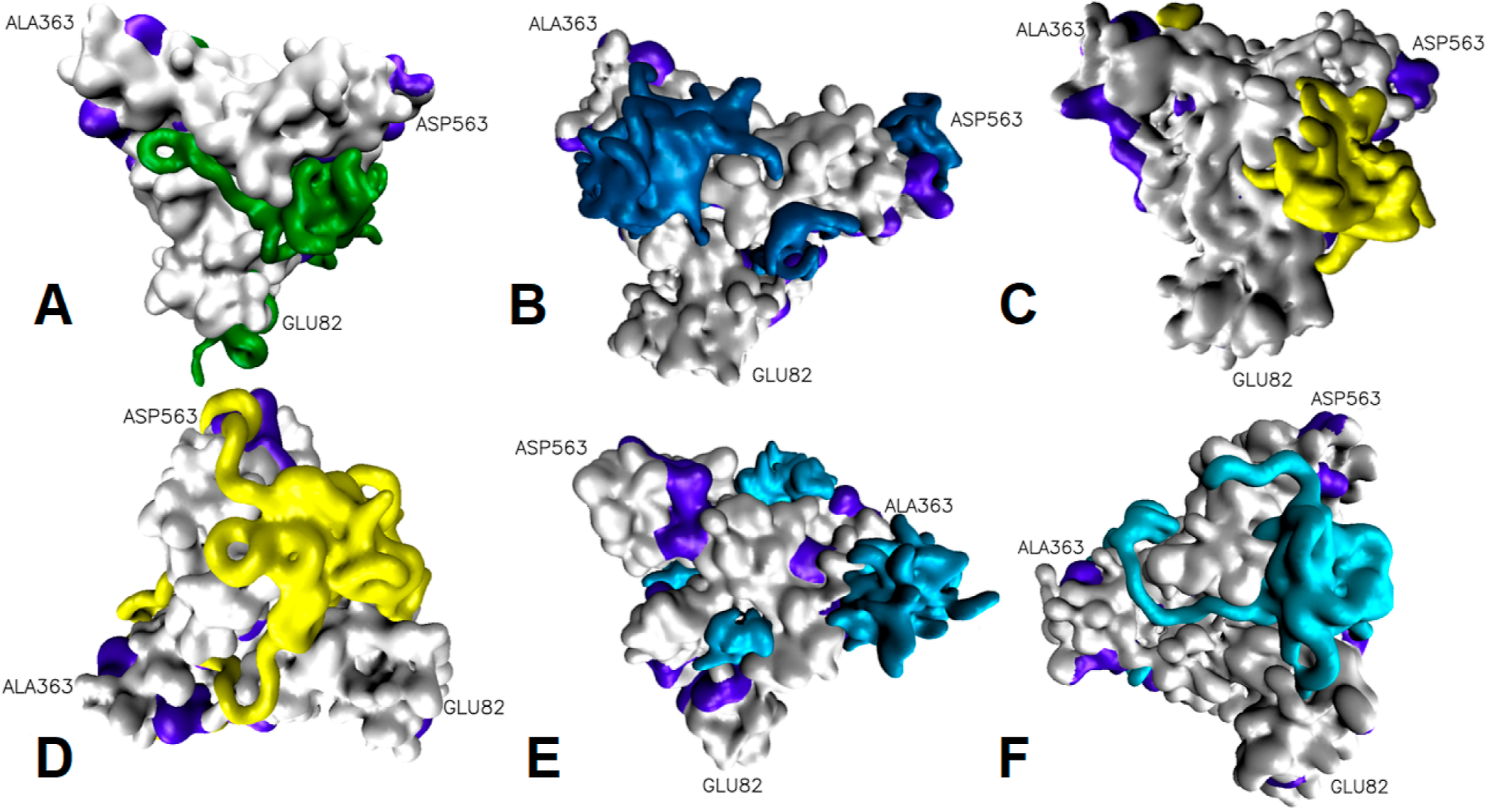
Select snapshots of trajectories at the end point of simulation, with the water removed for clarity. HSA is in gray with its APRs colored in violet. Glu82, Ala363, and Asp563 are labeled for orientation. Longer molecules wrap around the protein, while smaller molecules form clusters at the protein surface. (A) FAE O20. (B) Polysorbate 20. (C) PAA L2. (D) PAA O20. (E) Fatty alcohol ethoxylate (Brij) L2. (F) Fatty alcohol ethoxylate (Brij) O20. Gray, protein; violet, APR; green, FAE; dark blue, polysorbate; yellow, PAA; and light blue, fatty alcohol ethoxylate.

Visual inspection of the trajectories can also reveal the characteristics of the excipient–protein interaction. Qualitatively, compounds with a high PEG content, such as polysorbates or linear compounds with 20 PEG units, have a tendency to wrap around the protein while shorter ethoxylated compounds form localized, hemicellar clusters around a small number of residues (Figure 4). Unsurprisingly, of the simulations that showed little to no contact (such as phospholipids), little information can be gleaned from the nature of their interaction from the inspection of the arrangement in space. However, in the trajectories containing free arginine as an excipient, there is little evidence of continued sustained interaction, supporting the notion that its interaction is transient.

### Structure–Property Relationship

**Figure 5:**
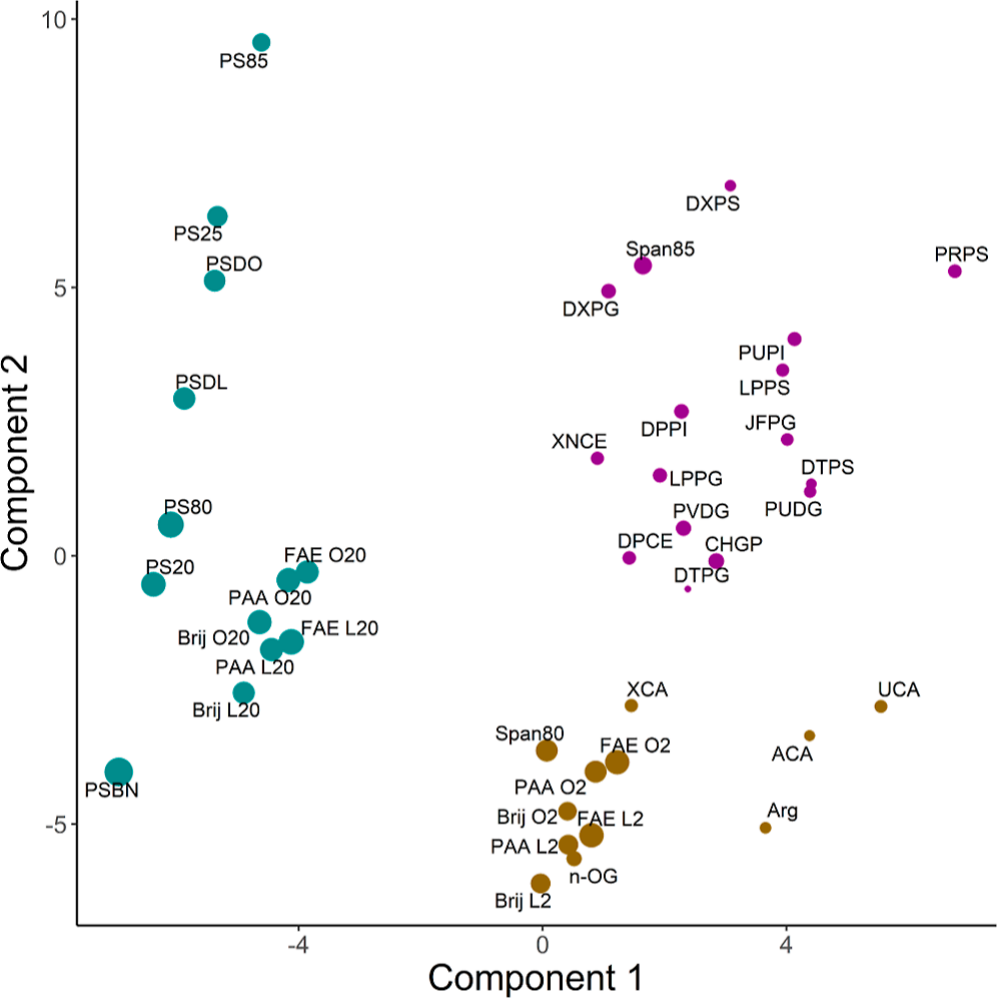
Distribution of the data in the latent variable space using Mordred descriptors as independent variables. Compounds are sized in proportion to the percentage decrease of APR SASA relative to the control and are colored according to their cluster. Cluster 1, teal; Cluster 2, gold; and Cluster 3, magenta.

The final PLS model of two components, validated with LOOCV, has an *R*^2^ value of 0.398 and a mean relative error of prediction of 0.077. To gain an understanding of the robustness of the data set and its validity in regression, the data set of excipient simulations was split 0.8/0.2 training data set/testing data set, and the *Q*^2^ was 0.344, with median root-mean-square errors of 4.10 and 4.37 nm^2^ for the training and test sets, respectively. These distributions of a measure of goodness of fit gives confidence that there is sufficient variation within the data set for its utility in a quantitative structure–property relationship application. Independently, a new model was constructed trained on all 41 instances to determine the importance of descriptors (and not to assess the predictive accuracy). There is a distinct divide between heavy molecules containing a relatively large amount of PEG that performed well in shielding APRs and thus improving stability and both smaller ethoxylated molecules and larger ones without any PEG (Figure 5). The PLS results show a clear demarcation between strongly interacting molecules and weak or noninteracting molecules and reveals physicochemical and structural differences between the two groups. Broadly, highly branched molecules and those with a high PEG content are within the well-performing cluster (cluster 1), while linear molecules and compounds with little to no PEG content are found within the other, broader cluster of poorly performing antiaggregation agents, with small ethoxylated compounds forming their own grouping along with fatty acids and arginine (cluster 2). The other poorly performing and/or PEG-lacking compounds make up a broadly dispersed cluster (cluster 3). There appears to be a moderate negative correlation between component 1 and the SASA of APRs.

The coordinates of Mordred variables in latent space, and their relation to compounds’ coordinates in the same space, can indicate the physicochemical forces involved in APR shielding. There are broadly similar but decidedly more scattered clusters within the variable space. Descriptors with a positive score in the second component and a negative score in the first component include those related to the number of oxygen atoms and the nature of their bonds, the number of heteroatoms, 5-membered rings, bond and atom polarizability, topological polar surface area, radius, and complexity, among others. Many of these descriptors have a clear relationship between them, such as the number of oxygen atoms and polar surface area. This specific example could indicate that there is a significant polar component that drives the shielding of the APRs from the solvent. The presence of the Bertz complexity score, a measure of molecular complexity and the distribution of heteroatoms, along with sp^3^ carbons bound to a single additional carbon, which in this context is either a terminal carbon or one within a furan ring, implies that greater APR shielding (and therefore enhanced stability) would be achieved by a branched compound with short aliphatic chains, a high degree of complexity and a broad distribution in space of a large number of heteroatoms. This is further supported by the lower impact on decreasing the SASA of APRs of compounds with a high alkyl chain content: phospholipids, Span 85 (three oleates), and glycerols all had little impact on the SASA of the APRs of HSA.

The poor performance of PS85 and Span 85 in particular could indicate an “activity cliff” relationship between APR shielding and aliphatic content, reflected in the positive coordinates in the latent space of descriptors concerning hydrocarbon content for both components in the region of cluster 2. Atom and bond polarizability are both influential in the formation of cluster 1 (negative value for component 1 and positive value for component 2), but mean polarizability is within cluster 2. This apparent discrepancy can be explained by the more highly mixed content of the well-performing ethoxylated compounds, having high molecular weights and their structures comprising polarizable and nonpolarizable bonds and atoms interspersed; conversely, those with a higher mean polarizability and less polarizable bonds and atoms have more chemically compact head groups, consisting of a small number of atoms with a high polarizability, and smaller hydrophobic tails, leading to a higher ratio of polarizability to molecular weight. This indicates that the shielding of APRs by excipients is better achieved by structures that have larger, but less extreme, polar characters like that which can be achieved by repeating ethoxylate units. Additionally, supporting this hypothesis is the position of the E-state descriptors that describe double-bonded oxygen (SdO and NdO) and the number of acid and base groups (nAcid and nBase). The mean van der Waals volume can also be found in this region, which could also be explained by the presence of bulky head groups in phospholipids, which are also found in this area as opposed to the lower occupational volume of PEG chains. Further evidence supporting this hypothesis is the positions and relative importance of the topological radius, topological polar surface area, and the number of rotatable bonds. Qualitatively, many of these qualities can be found in compounds with high PEG content, and the data reflect the preferential interaction to APRs of polysorbate species and linear compounds with high PEG content.

The impact of PEG content on increasing protein–excipient interaction could be due to entropic effects; longer ethoxylated compounds would have a greater loss of entropy upon burial, as the hydrophobic tail is more readily buried within the hydrophilic head groups. This is indicated by the SASA differential observed between linear PEGylated compounds of differing PEG length. Compounds with 20 PEG units typically have a greater effect on the reduction of the SASA than those with 2 PEG units, despite these simulations having approximately the same quantity of EO beads but different numbers of molecules. These compounds also appear to more readily form intermolecular clusters, independent of the protein, that are reminiscent of micelles. These behaviors are also exhibited by the polysorbate compounds. Together, these behaviors indicate a strong influence of entropy on the interaction between protein APRs and stabilizing excipients. Qualitatively, long PEG chains have a greater tendency to occupy channels on the surface of the protein; these valleys are lined with polar residues, but nonpolar residues typically make up the “floor”. Thus, the larger PEG chains are able to make a large number of polar–polar contacts to reduce the SASA, and their intermediate polarity as Martini beads allows them to occupy these surface channels without a prohibitive degree of repulsion.

## Discussion

MD simulations have been employed to investigate the efficacy of excipients as antiaggregation agents and probe the importance of APR interaction as a mechanism for the prevention of biotherapeutic aggregation. The APRs of HSA have been identified using an experimentally derived aggregation propensity score via the Aggrescan web server, and the propensity of an excipient molecule to interact with both the APR and the entirety of HSA has been utilized as an effective demonstration of the APR-shielding mechanism of the arrest of aggregation. Generally, molecules with a high degree of PEG content reduced the SASA of APRs, with little impact from any differences in hydrophobic content within ethoxylated compounds and almost no change between HSA and compounds with high hydrophobic content that lack PEG. This suggests that the interaction between the protein and the polar PEG chains that constitute the headgroup is driving the overall increase in interaction, a finding that is supported by the literature.^84−86^ As protein aggregation is driven primarily by hydrophobic interactions with contributions from polar interactions,^13^ this could indicate that the increase in polar interaction is contributing to the overall stability of the protein by tipping the scales in the direction of polar interaction and making the hydrophobic destabilizing interactions less significant overall. This notion is further supported by the near total lack of interaction between HSA and the naturally occurring phospholipids under study; compounds with the largest hydrophobic tails and comparatively small head groups have little interaction. Similarly, Spans (in essence, polysorbates lacking PEG) and polysorbates with more than one fatty acid ester, such as PS85 and PS25, perform worse in terms of APR SASA shielding than PSBN, a branched compound with little nonpolar content and a high proportion of PEG content. This also implies that an increase in molecular weight is not sufficient to increase antiaggregation activity, further supported by the absence of impact of molecular weight as a descriptor or as a factor within a descriptor within PLS. Together with the observation that larger molecules have a tendency to wrap around HSA, this could imply that the headgroup initiates the interaction before recruiting the tail in wrapping around more hydrophobic areas of the protein. It can also be seen that longer interacting compounds are making end-to-end contact with each other within a shallow channel on the protein surface (Figure 4A,F). This is reminiscent of binding behavior observed in crystallographic binding studies with short- and medium-chain fatty acids.^87^ Polysorbate 20 and 80 specifically have also been found to interact with HSA, albeit weakly,^55^ which has also been reproduced in this study. The use of Aggrescan, which calculates the average aggregation propensity of sequences based on experimentally derived values for each amino acid in the context of the formation of amyloid plaques,^88^ as the sole indication of APRs could be improved by the inclusion of other methods in a comparative way. One such method would be spatial aggregation propensity (SAP),^17^ which considers whether residues are either exposed to the solvent or buried. Using additional methods to flag APRs would ensure a comprehensive approach in finding areas of the protein that are significant in the aggregation process and therefore improve the robustness of the model.

The lack of interaction between HSA and every phospholipid under study is surprising, given HSA’s role in transporting fatty acids^89^ and cholesterols^90^ in circulation and studies of its interaction with phospholipid membranes.^91,92^ However, the concentrations of lipid used in the membrane studies are typically significantly greater than those of excipients in the present study; typically, these are millimolar as opposed to 0.1% w/w, which results in concentrations in the range of 0.10–0.18 mM. For all phospholipids with at least 12 carbons in their fat chains, this concentration range is above the critical micellar concentration (CMC);^93^ the lack of differentiation along the CMC of the compounds under study implies that it is not of critical importance in this context; heavy phospholipids above the CMC perform equally poorly to lighter phospholipids below it, and so, other factors are more significant in determining the extent of interaction. This concentration of 0.1% w/w was chosen to emulate industrial conditions for primarily surfactant excipients used in biotherapeutic stabilization formulations; for other excipients such as those that include sugar residues and arginine, their working concentrations are typically higher.

One limitation of this study is the modeling of polysorbates as homogeneous additives, when in reality, they are typically a heterogeneous mixture that contains byproducts with ranges of differences in aliphatic and PEG chain lengths and number.^94,95^ This is particularly of note as the heterogeneity of polysorbate commercial products impacts their ability to prevent aggregation; polysorbate fractions vary in their performance in this context.^96^ Therefore, it could be prudent to model polysorbate as a heterogeneous mixture; to maintain concentrations that are industrially relevant, this would likely require the modeling of extremely large systems.

Validation could also be provided in the characterization of excipient effects on protein stability, by monitoring changes in aggregate size, protein secondary and tertiary structures, and biological activity assays. However, the stability of HSA and its own use as an antiaggregation agent^97^ would make reliably inducing (and measurably arresting or preventing) aggregation challenging. This points to a need for a protein-independent model, which would be most easily developed by modeling one or more different therapeutically relevant proteins, ideally with their own stability issues, such as insulin or the binding fragment of an antibody. Additionally, validation of the PLS model can be increased by the introduction of more simulation data, which can be either included in the predictive model or excluded from it and used as a validation test set.

By using techniques to explore latent variable space and probe the physicochemical properties of each excipient and how they correlate with antiaggregation activity, hypotheses on the design of novel excipients with greater APR SASA shielding, and therefore improved performance as antiaggregation agents, can be postulated. Particularly, variable importance in projection (VIP) plots are used in feature selection in drug design^98^ and are a useful tool in investigating the structure–property relationship within a PLS model by indicating the critical descriptors that explain the maximal variance in both dependent and independent variables. An optimized excipient would be a large, branched compound which is highly polar (i.e., with several oxygen atoms) and also of some hydrophobic character. Practically, this could be achieved by the incorporation of multiple PEG chains into the excipient design around a central scaffold and at least one aliphatic chain. This is broadly descriptive of a polysorbate compound, and this is perhaps unsurprising considering their performance, but it also indicates that there is chemical space that is underutilized by the current antiaggregation excipient design paradigm. It implies that the exact degree of hydrophobic content is not significant, provided that there is some present in a localized area in order to provide amphiphilic character to the excipient. The findings suggest that perhaps a compound with a lower molecular weight and a higher number of short branches would be more effective in APR shielding than heavier compounds with a small number of large chains. Such a compound might be achieved by the utilization of an oligopeptide or dendrimer central scaffold, functionalized by multiple short-chain ethoxylation and fatty acid groups on termini and side chains.

## Conclusions

The coarse-grained modeling of HSA with a series of cosolutes has revealed structural and physicochemical features that are highly influential to the prevention of aggregation via APR shielding. Broadly, ethoxylated compounds had the greatest performance as APR-shielding antiaggregation agents, and polysorbate species specifically were the highest performing class. Branched compounds tended to make greater contact with APRs, particularly those with PEG chains, while phospholipids and fatty acids performed very poorly in shielding APRs from solvent and thereby preventing aggregation. The use of dimensionality reduction coupled with physicochemical descriptors has revealed structural features that are key to optimizing protein–excipient interaction. The overall weight of aliphatic chains does not appear to influence the performance of antiaggregation agents, provided that some is present. The significance of polarity, polarizability, and polar heteroatom content in predicting HSA interaction also suggests that the interaction between APRs and excipients is driven by polar interactions to a significant degree. The quantitative model would be well-supported by future endeavors that elucidate free energy differences, provide validation via wet-lab work or atomistic MD, and move away from a singular protein to develop a more widely applicable, predictive model to aid in computational excipient design and improve the stability of biotherapeutic formulations.

## Data Availability

SASA data can be found at the GitHub repository (https://github.com/TobyEdwardKing/Excipient-Optimisation), as can the descriptor data and the compounds’ SMILES. Gromacs is a freely available software package for molecular dynamics, and details on its installation can be found on their Web site: www.gromacs.org. The following packages in R were used in the extraction of data, development of the model, and generation of figures: Peptides, scico, tidyverse, ggpubr, pls, webchem, rcdk, and vip. All are freely available from the CRAN repository. Mordred, a Python package, was used to extract quantitative structure–property activity information from SMILES structures, in conjunction with rdkit, numpy, and pandas, and all can be retrieved freely. Some molecular dynamics graphics were created with VMD, freely available from http://www.ks.uiuc.edu/Research/vmd.

## Supporting Information Available

Bash script for molecular dynamics simulations and their subsequent analysis, parameter files for excipient compounds, structural files for excipient compounds, structural depictions of excipients under study, R script detailing PLS model, raw data in an archive, Mordred data input for the PLS model, and data table of excipient SMILES and associated data (PDF)
