# Supplementary material for: Optimizing Excipient Properties to Prevent Aggregation in Biopharmaceutical Formulations
Source: J Chem Inf Model. 2023 Dec 19;64(1):265–75. doi: 10.1021/acs.jcim.3c01898 (PMC10777730; doi:10.1021/acs.jcim.3c01898)
Supplement: Supplementary file 1 — ci3c01898_si_001.pdf [file ci3c01898_si_001.pdf]

Supporting Information: Optimising excipient properties to prevent aggregation in biopharmaceutical formulations

Toby E. King,[†] James R. Humphrey,[‡] Charles A. Laughton,[†] Neil R. Thomas,[¶] and Jonathan D. Hirst[*,§]

†*Biodiscovery Institute, School of Pharmacy, University Park, Nottingham, NG7 2RD, UK*

‡*Croda Europe Ltd, Cowick Hall, Snaith, DN14 9AA, UK*

¶*Biodiscovery Institute, School of Chemistry, University Park, Nottingham, NG7 2RD, UK*

§*School of Chemistry, University Park, Nottingham, NG7 2RD, UK*

E-mail: jonathan.hirst@nottingham.ac.uk

Structural representations in MARTINI

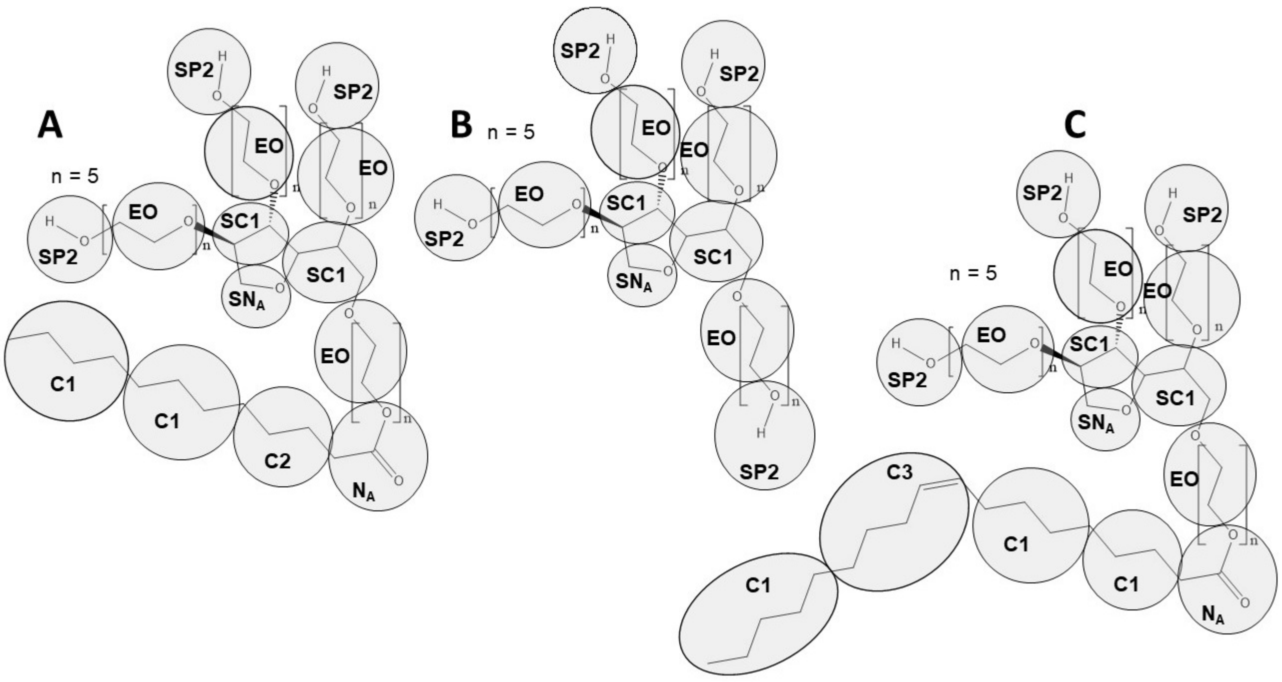

Figure S1: Structural representations of A: polysorbate monolaurate (PS20); B: polyoxyethylene sorbitan (PSBN) and C: polysorbate monooleate (PS80). MARTINI representations are superimposed.

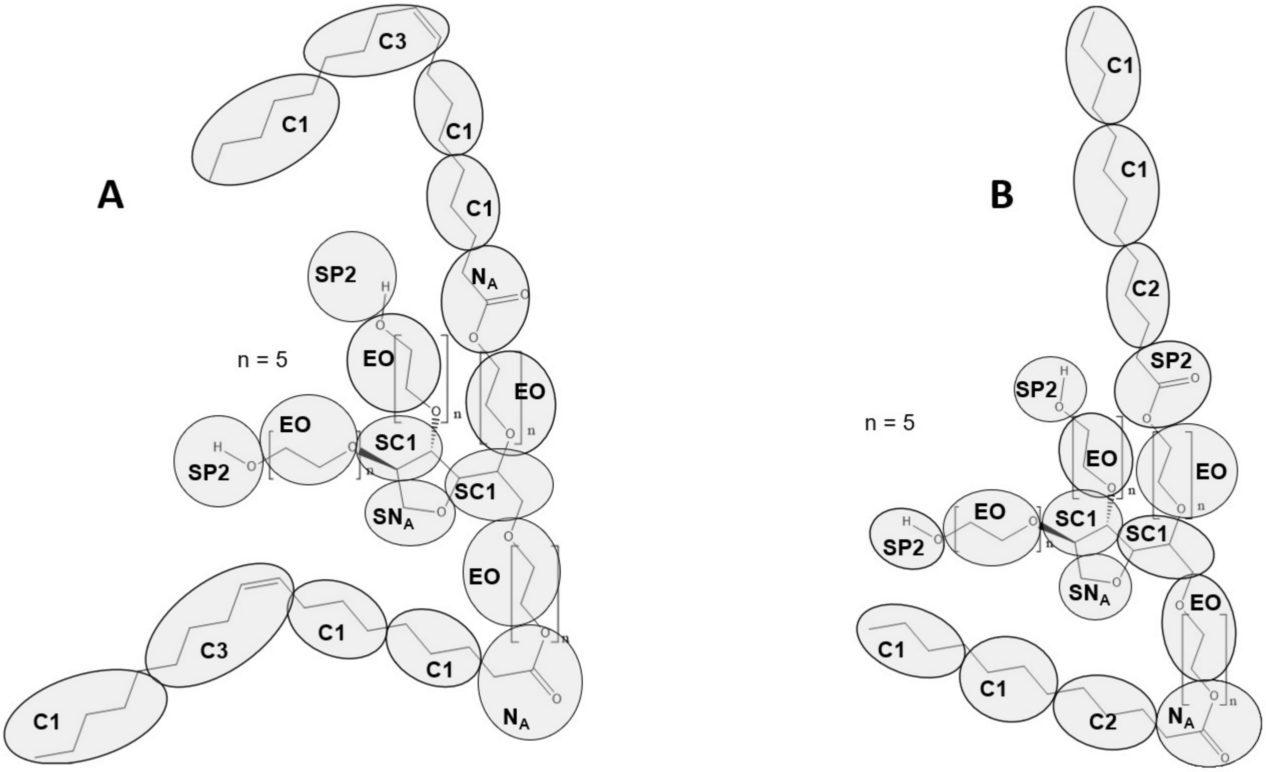

Figure S2: Structural representations of A: polysorbate dioleate (PSDO) and B: polysorbate dilaurate (PSDO). MARTINI representations are superimposed.

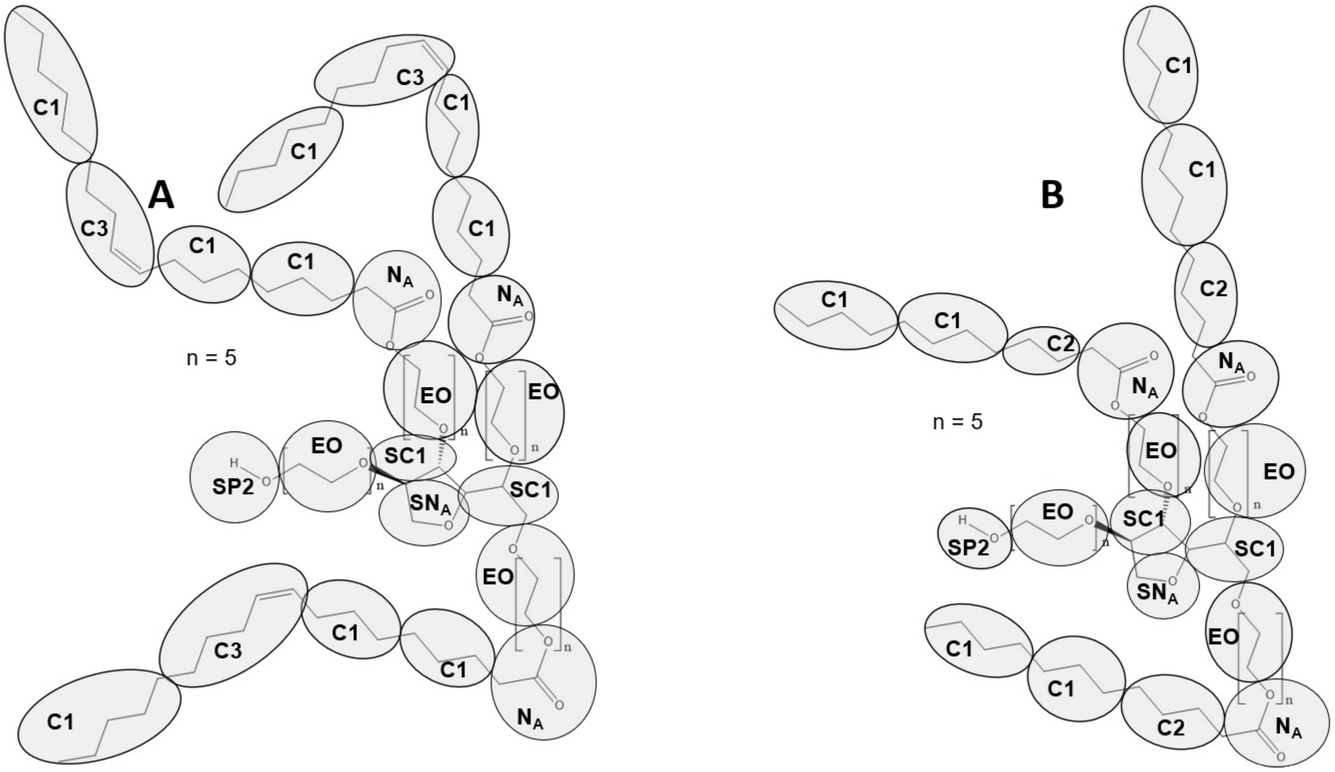

Figure S3: Structural representations of A: polysorbate trioleate (PS85) and B: polysobate trilaurate (PS25). MARTINI representations are superimposed.

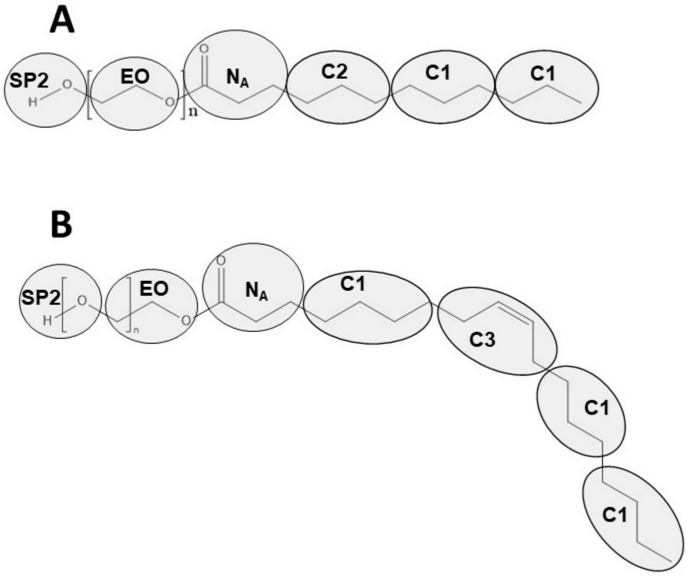

Figure S4: Structural representations of fatty acid ethoxylates. A: fatty acid ethoxylate laurate n (FAE Ln). B: fatty acid ethoxylate oleate n (FAE On). MARTINI representations are superimposed.

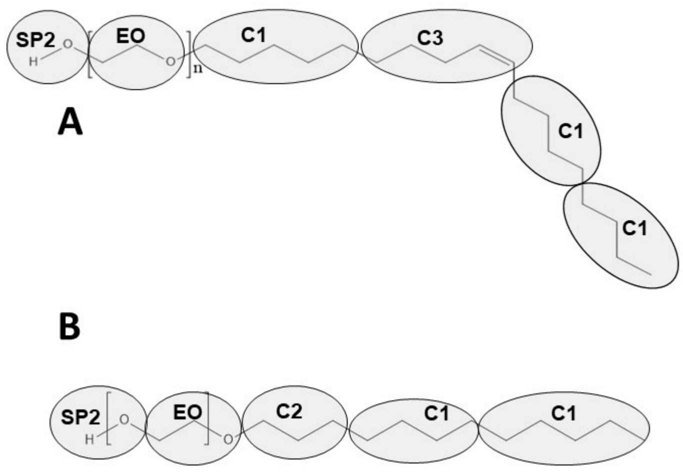

Figure S5: Structural representations fatty alcohol ethoxylates, or Brij compounds. A: fatty alcohol ethoxylate laurate n (Brij Ln) and B: fatty alcohol ethoxylate oleate n (Brij On). MARTINI representations are superimposed.

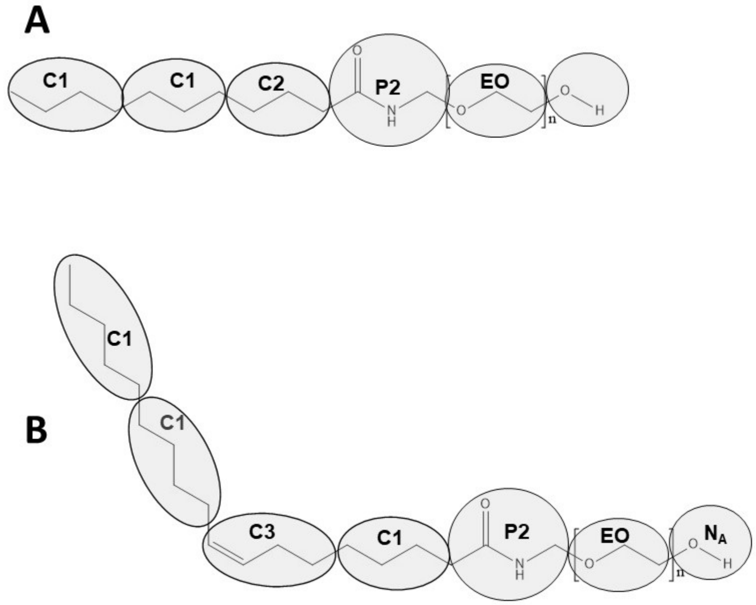

Figure S6: Structural representations of PEG alkyl amides. A: PEG alkyl amide laurate$n$ (PAA Ln). B: PEG alkyl amide laurate oleate$n$ (Brij On). MARTINI representations are superimposed.

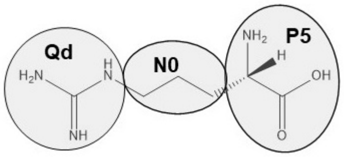

Figure S7: Structural representation of arginine with its MARTINI representation superimposed.

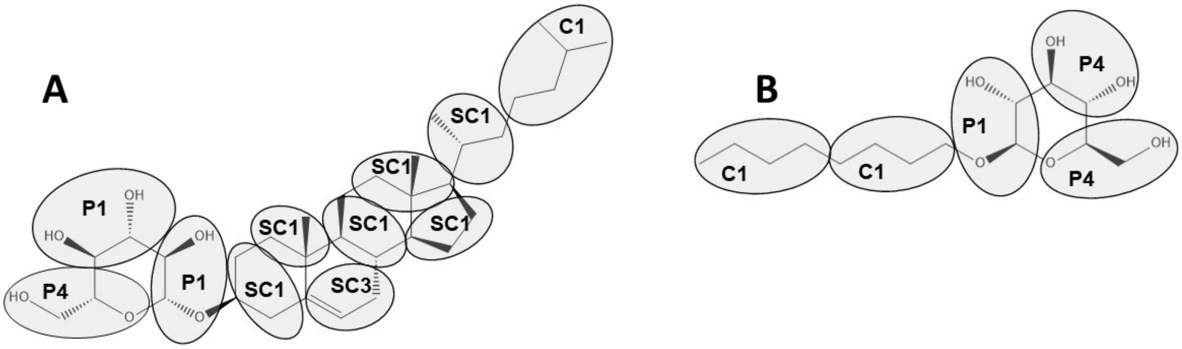

Figure S8: Structural representation of glucosides. A: cholestyryl glucopyranoside (CHGP). B: *n*-octyl glucoside. MARTINI representations are superimposed.

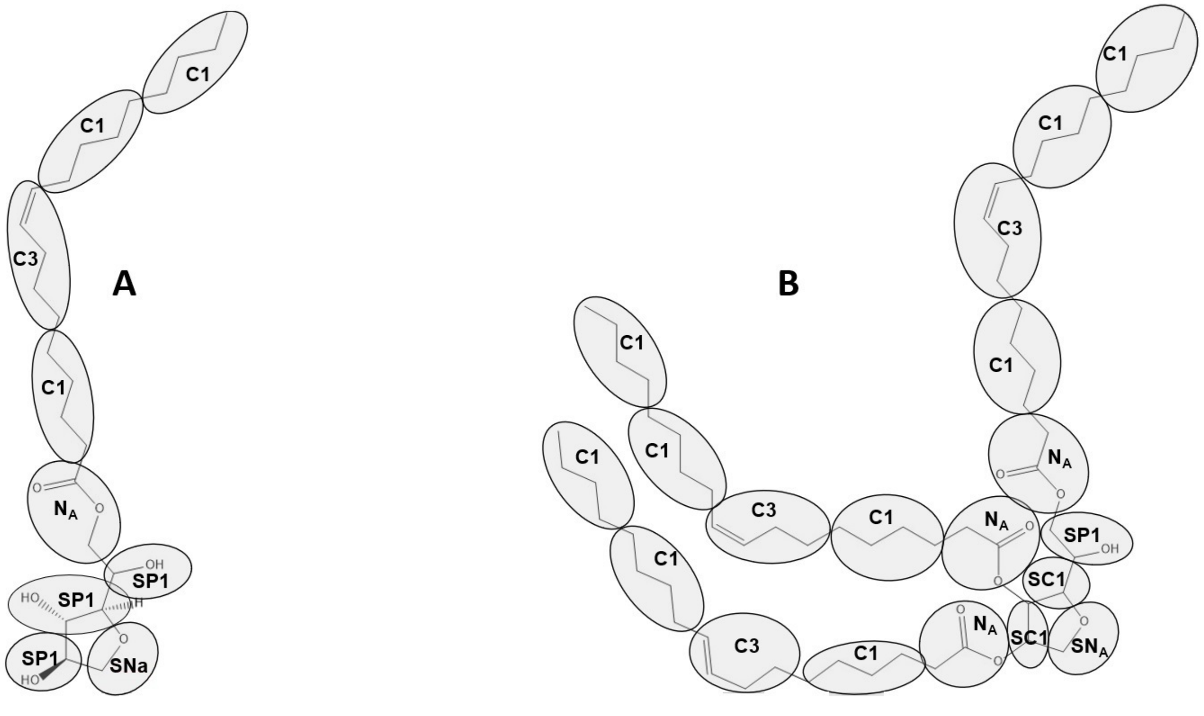

Figure S9: Structural representation of Spans. A: Span 80. B: Span 85. MARTINI representations are superimposed.

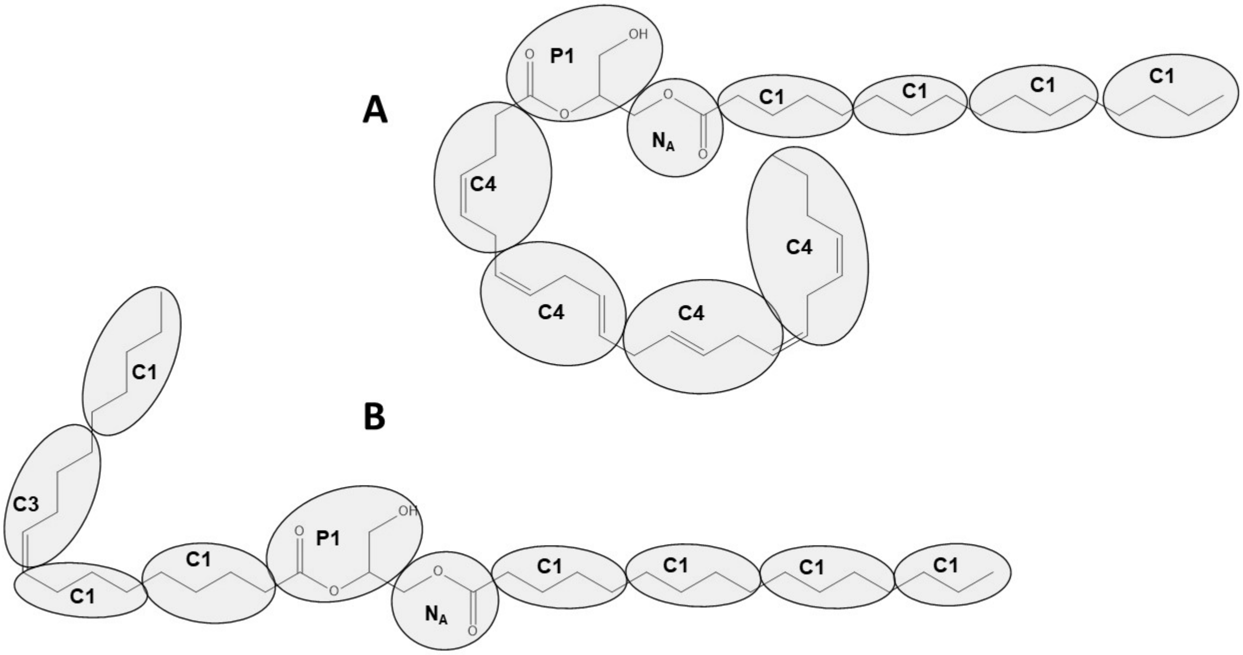

Figure S10: Structural representation of diacylglycerols. A: C16:0/22:6 (PUDG). B: C18:1C12:0 (PVDG). MARTINI representations are superimposed. Representations retrieved from the parameter repository at cgmartini.nl.

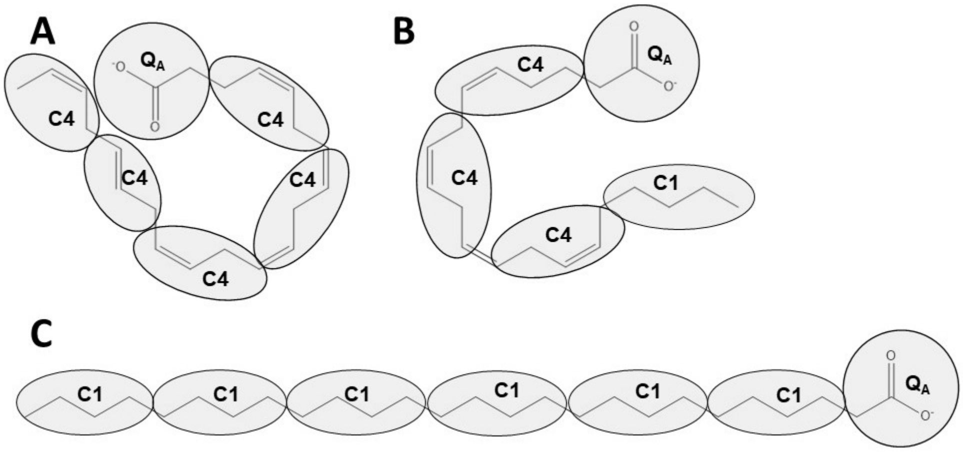

Figure S11: Structural representation of fatty acids. A: docosahexaenoic acid (UCA). B: arichadonic acid (ACA). C: hexacosanoic acid (XCA). MARTINI representations are superimposed. Representations retrieved from the parameter repository at cgmartini.nl.

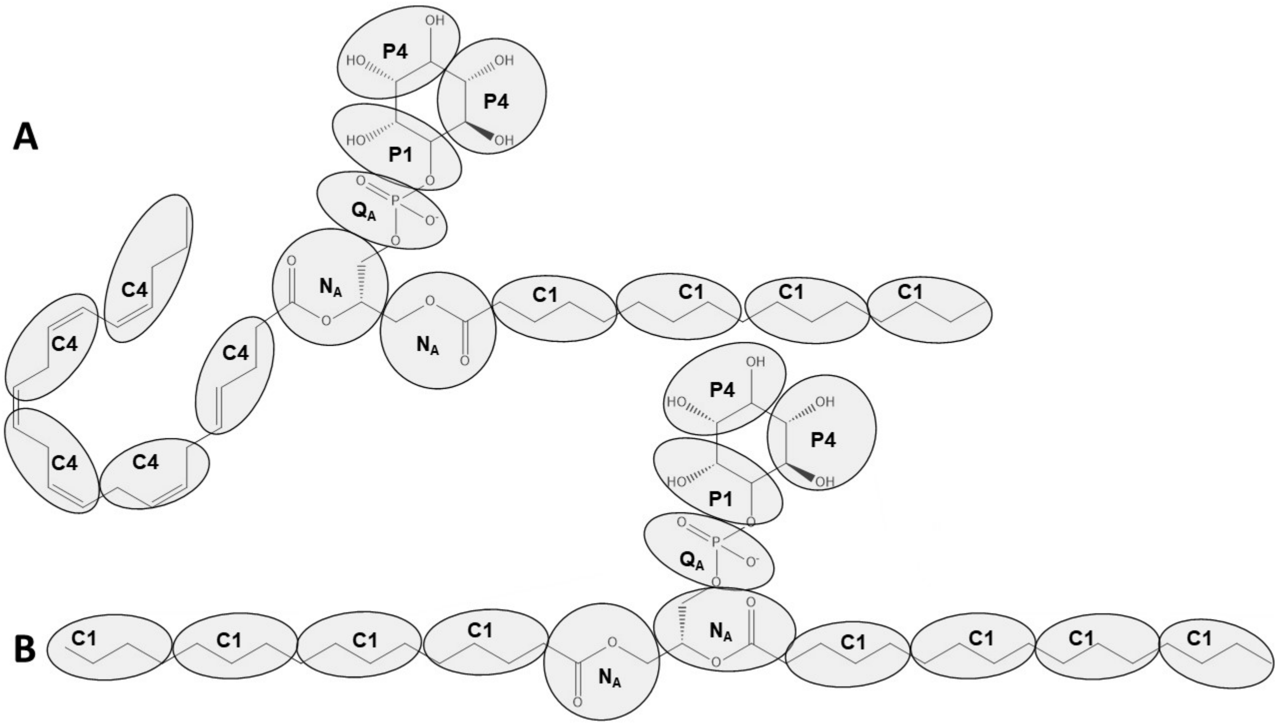

Figure S12: Structural representation of phosphatidylinositols. A: 1-palmitoyl-2-docosahexaenoyl phosphatidylinositol (PUPI). B: dipalmitoyl/distearoyl phosphatidylinositol (DPPI). MARTINI representations are superimposed. Representations retrieved from the parameter repository at cgmartini.nl.

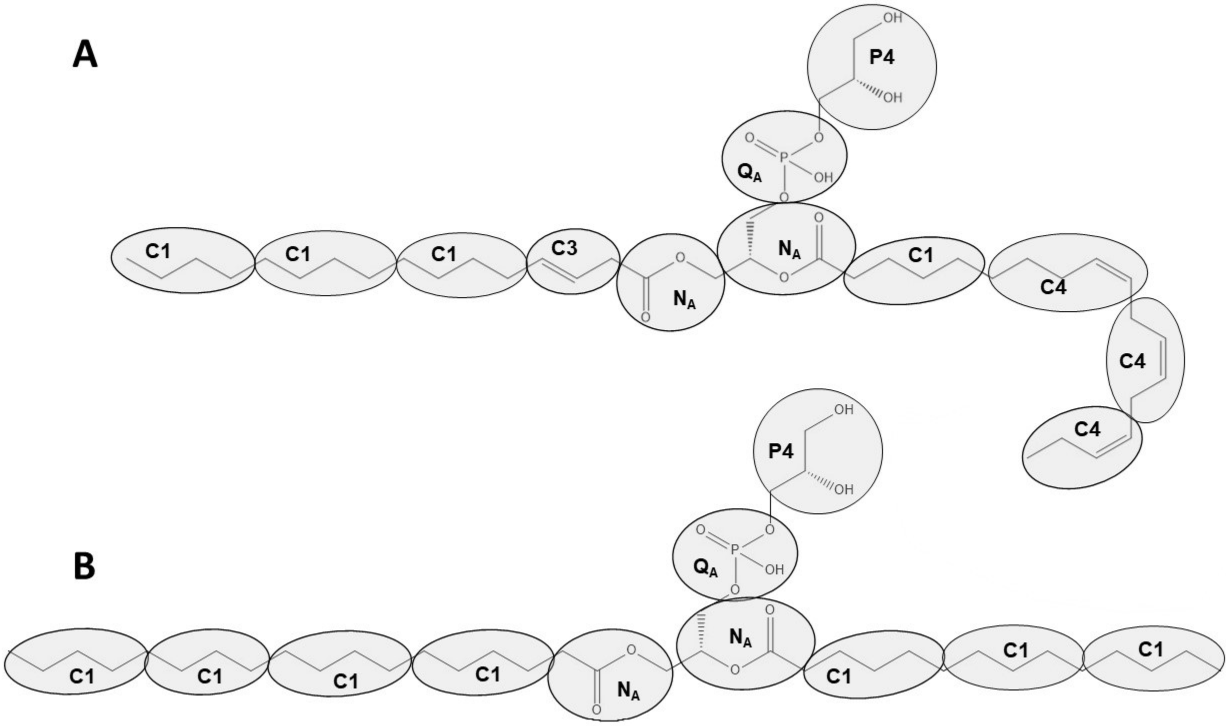

Figure S13: Structural representation of phosphatidylglycerols. A: trans-3-hexadecenoic acid/linolenoyl tailed phosphatidylglycerol (JFPG). B: 1-myristoyl/2-stearoyl tailed phosphatidylglycerol (LPPG). MARTINI representations are superimposed. Representations retrieved from the parameter repository at cgmartini.nl.

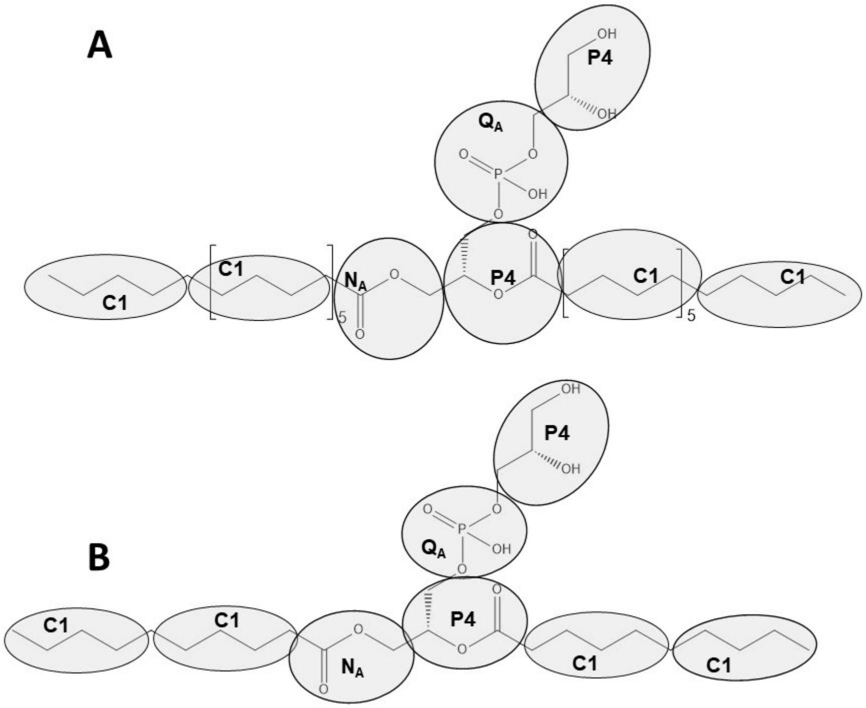

Figure S14: Structural representation of phosphatidylglycerols. A: dihexacosanoyl tailed phosphatidylglycerol (DXPG). B: didecanoyl tailed phosphatidylglycerol (DTPG). MARTINI representations are superimposed. Representations retrieved from the parameter repository at cgmartini.nl.

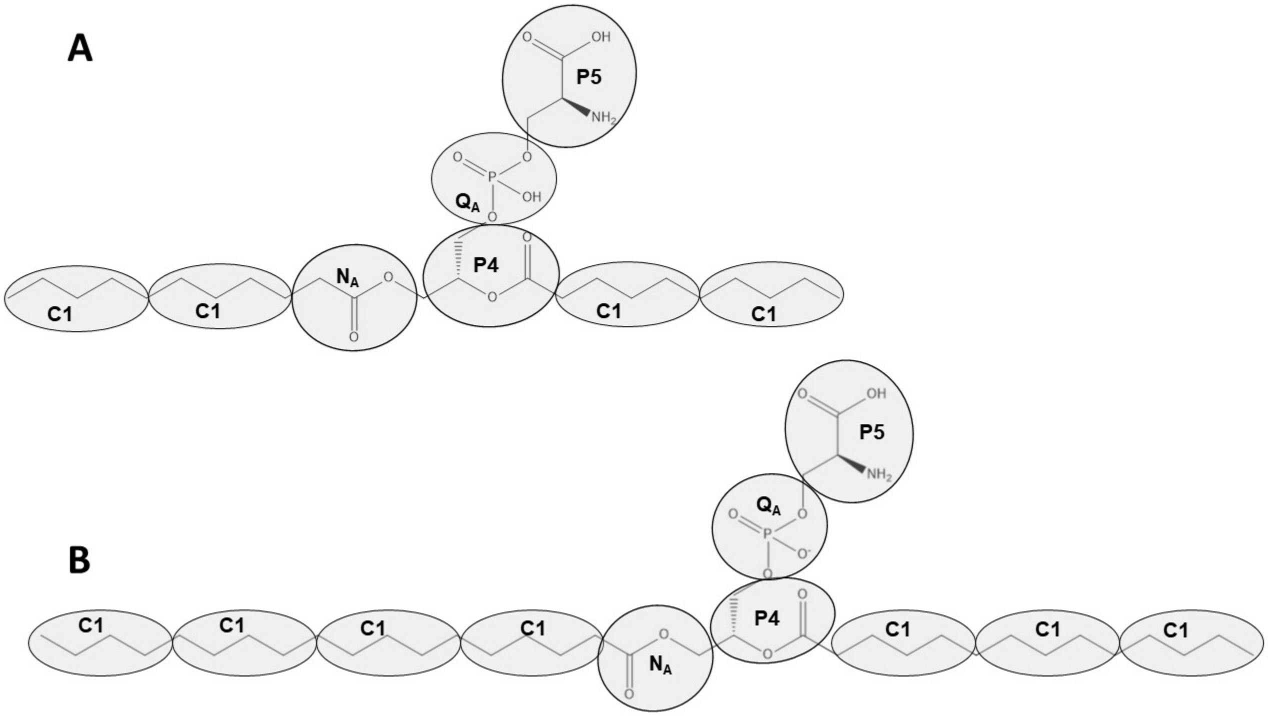

Figure S15: Structural representation of phosphatidylserines. A: didecanoyl tailed phosphatidylserine(DTPS). B: 1-myristoyl-2-stearoyl tailed phosphatidylserine (LPPS). MARTINI representations are superimposed. Representations retrieved from the parameter repository at cgmartini.nl.

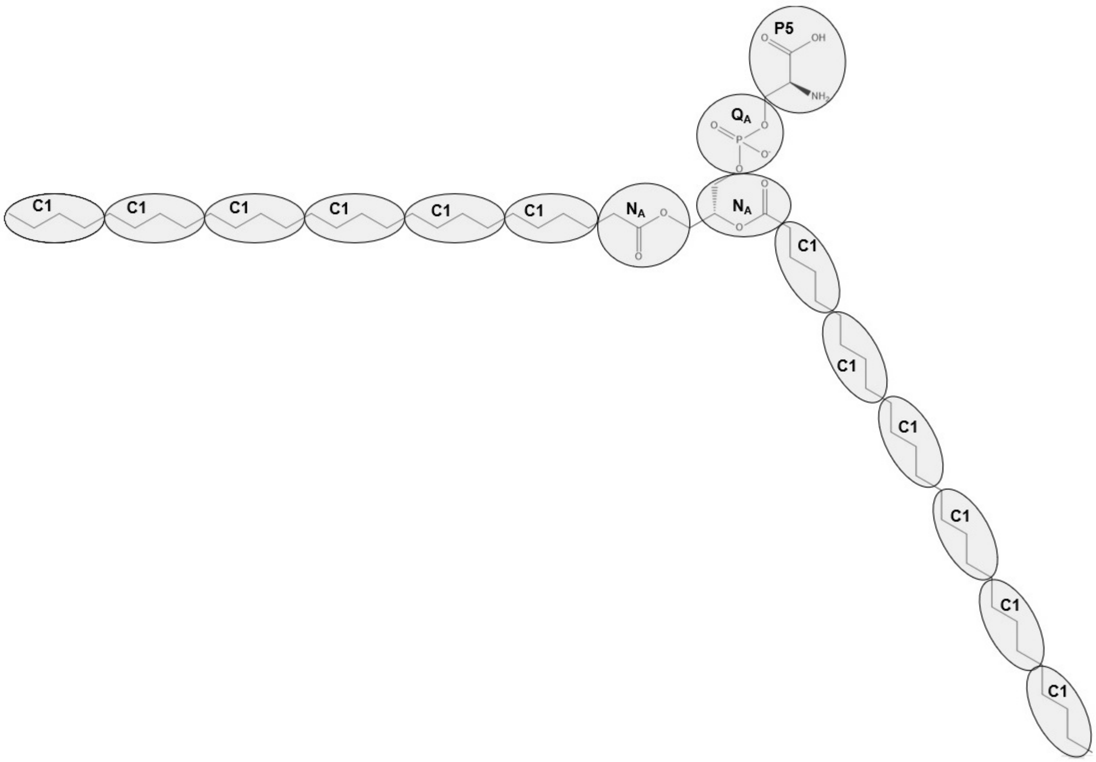

Figure S16: Structural representation of dihexacosanoyl tailed phosphatidylserine (DXPS). MARTINI representations are superimposed. Representations retrieved from the parameter repository at cgmartini.nl.

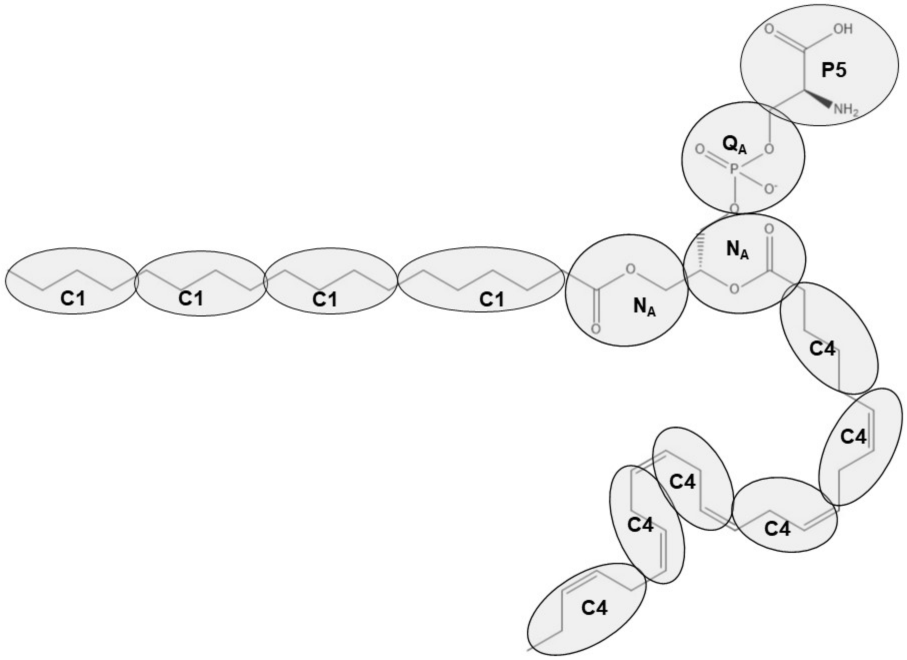

Figure S17: Structural representation of ditetracosahexaenoic acid tailed phosphatidylserine (DRPS). MARTINI representations are superimposed. Representations retrieved from the parameter repository at cgmartini.nl.

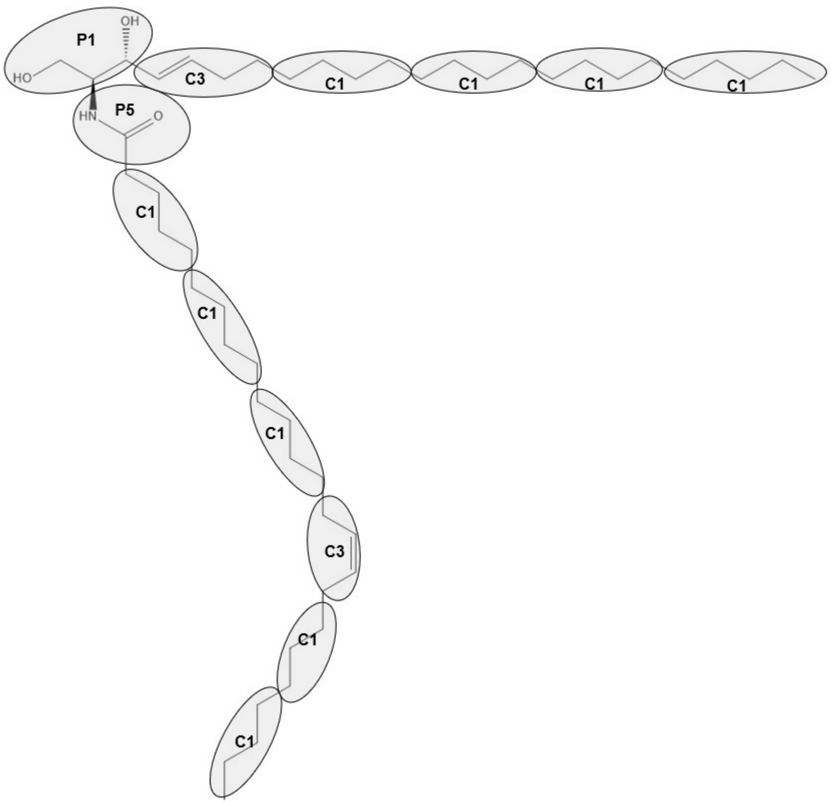

Figure S18: Structural representation of C24:1/C24:1 tailed ceramide (XNCE). MARTINI representations are superimposed. Representations retrieved from the parameter repository at cgmartini.nl.

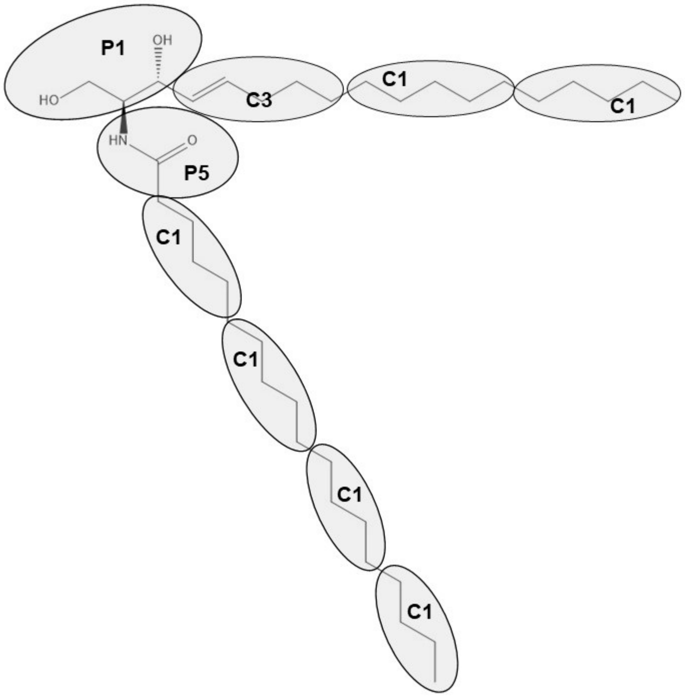

Figure S19: Structural representation of N-stearoyl-D-erythro tailed ceramide (DPCE). MARTINI representations are superimposed. Representations retrieved from the parameter repository at cgmartini.nl.

Equilibration and convergence

In order to assess equilibration, the total energy of the system as a function of time was transformed into a Z score by subtracting the mean and dividing by the standard deviation. Each trajectory was divided into 100 equal segments, and the difference in Z-score between the first and second halves of a trajectory comprising a given number of segments was measured after discarding a progressively greater number of segments of the simulation, starting with no segments discarded and concluding with 99 segments discarded. Equilibrium was considered to be reached when the difference in Z-score reached a minimum. Using the total energy as a metric, the latest point at which this occurred was 75%. This was also the case when considering, as a metric, the frequency of occurrence of any excipient molecule being closer than or equal to 0.6 nm (a contact). The convergence of each simulation was estimated in a similar approach to equilibration; the trajectories were split into segments as with equilibration, and the difference in Z-score between the first and second half was measured after including a progressively larger percentage of the trajectory, starting with a single segment and ending with 100 segments. A trajectory was considered to be converged if the difference between the two halves tended towards zero, and all simulations were converged after the 76th segment when using both total energy and the number of protein-excipient contacts as a metric.

Human serum albumin and native binding

As HSA contains binding sites for fatty acids which have been found to be involved in polysorbate binding,[S1] it is necessary to consider whether the interaction is exhibited with APRs, or with residues involved in binding. These residues are involved in fatty acid binding by interacting directly with a fatty acid carboxylate moiety, or by their collective formation of a hydrophobic binding pocket (80 residues), or both, and have been characterised previously.[S2] Some of these residues are also predicted to reside within an APR (figure S21).

A residue within an APR is less likely to be found within a hydrophobic cavity than elsewhere, as its proportion of APR-inhabiting residues (13.7%) is lower than the overall figure of 25.4%. Not only that, but only 21.5% of APR residues are also in the binding pocket. Generally, the residues that are both in the binding pocket and involved in head binding have lower SASA on average than those that aren't, a difference that is more pronounced if those residues are predicted to be present in an APR (figure S20). However, when residues are involved in all three, their SASA is typically much higher, but there are only two that fit these criteria (K525 and R117) and so this distinction cannot be considered statistically important. On average, the residues with the lowest SASA are those that are found in the cavity that are also predicted to be APRs, followed by those that solely comprise the cavity. Together, although the interaction of hydrophobic cavity residues with excipient molecules is responsible for some degree of the overall interaction, the majority of SASA reduction of APRs takes place external to the pocket.

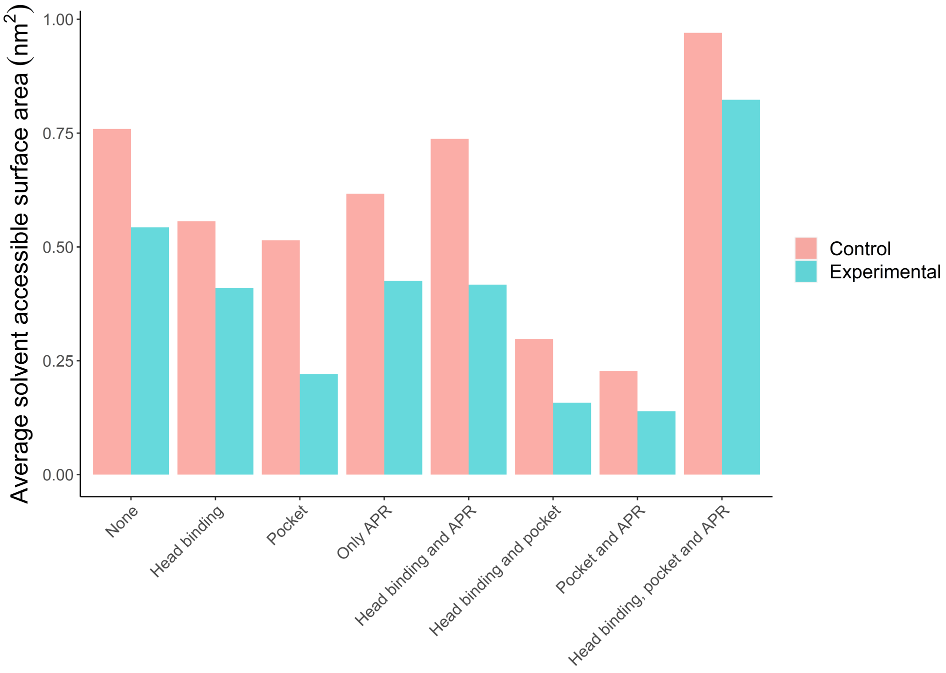

Figure S20: Average SASA of each residue, grouped by whether it is found within the hydrophobic binding pocket, would usually be involved in carboxylate head interaction, is in an APR, or any combination thereof.

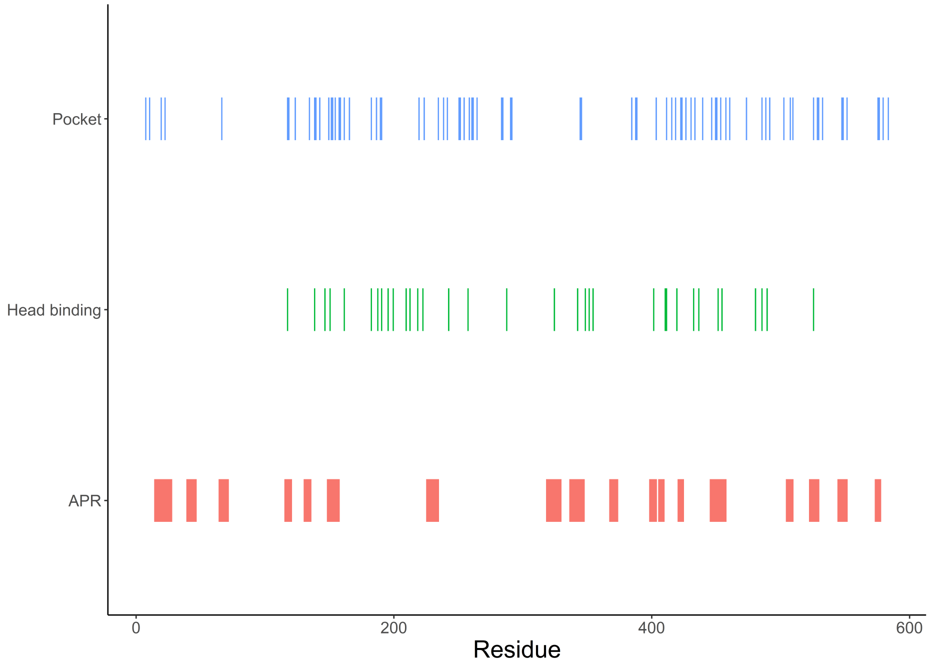

Figure S21: Residency of each residue in an aggregation-prone region, an endogenous fatty acid head group-binding residue, and/or a hydrophobic binding pocket. Head group binding and hydrophobic pocket residues are from Curry *et al.*[S2]

References

(S1) Garidel, P.; Hoffmann, C.; Blume, A. A thermodynamic analysis of the binding interaction between polysorbate 20 and 80 with human serum albumins and immunoglobulins: A contribution to understand colloidal protein stabilisation. *Biophys. Chem.* **2009**, *143*, 70–78.

(S2) Curry, S.; Mandelkow, H.; Brick, P.; Franks, N. Crystal structure of human serum albumin complexed with fatty acid reveals an asymmetric distribution of binding sites. *Nat. Struct. Mol. Biol.* **1998**, *5*, 827–835.
